# The Deubiquitinase USP29 Promotes SARS-CoV-2 Virulence by Preventing Proteasome Degradation of ORF9b

**DOI:** 10.1128/mbio.01300-22

**Published:** 2022-05-31

**Authors:** Wenying Gao, Liuli Wang, Xiaohui Ju, Simin Zhao, Zhaolong Li, Manman Su, Jiancheng Xu, Peihui Wang, Qiang Ding, Guoyue Lv, Wenyan Zhang

**Affiliations:** a Center of Infectious Diseases and Pathogen Biology, Institute of Virology and AIDS Research, Key Laboratory of Organ Regeneration and Transplantation of The Ministry of Education, The First Hospital of Jilin University, Changchun, China; b Department of Hepatobiliary and Pancreatic Surgery, The First Hospital of Jilin University, Changchun, Jilin, China; c College of Medicine, Jilin University, Changchun, China; d School of Medicine, Tsinghua University, Beijing, China; e Department of Laboratory Medicine, First Hospital of Jilin University, Changchun, China; f Key Laboratory for Experimental Teratology of Ministry of Education and Advanced Medical Research Institute, Cheeloo College of Medicine, Shandong University, Jinan, China; University of Calgary

**Keywords:** SARS-CoV-2, ORF9b, degradation, USP29, deubiquitination

## Abstract

Ubiquitin signaling is essential for immunity to restrict pathogen proliferation. Due to its enormous impact on human health and the global economy, intensive efforts have been invested in studying severe acute respiratory syndrome coronavirus 2 (SARS-CoV-2) and its interactions with hosts. However, the role of the ubiquitin network in pathogenicity has not yet been explored. Here, we found that ORF9b of SARS-CoV-2 is ubiquitinated on Lys-4 and Lys-40 by unknown E3 ubiquitin ligases and is degraded by the ubiquitin proteasomal system. Importantly, we identified USP29 as a host factor that prevents ORF9b ubiquitination and subsequent degradation. USP29 interacts with the carboxyl end of ORF9b and removes ubiquitin chains from the protein, thereby inhibiting type I interferon (IFN) induction and NF-κB activation. We also found that ORF9b stabilization by USP29 enhanced the virulence of VSV-eGFP and transcription and replication-competent SARS-CoV-2 virus-like-particles (trVLP). Moreover, we observed that the mRNA level of USP29 in SARS-CoV-2 patients was higher than that in healthy people. Our findings provide important evidence indicating that targeting USP29 may effectively combat SARS-CoV-2 infection.

## INTRODUCTION

The severe acute respiratory syndrome coronavirus 2 (SARS-CoV-2) genome encodes 4 structural (S, E, M, and N), 16 nonstructural (nsp1-16), and up to 11 accessory proteins (3a–3d, 6, 7a, 7b, 8, 9b, 9c, and 10). Most studies have focused on discovering the functions of structural and nonstructural viral proteins; however, the roles of accessory proteins in SARS-CoV-2 pathogenesis are still not completely understood. Accumulating evidence demonstrates that accessory proteins are critical for virus–host interactions and pathogenesis (1). The accessory protein ORF9b of SARS-CoV-2 was recently reported to antagonize type I and type III interferon (IFN) production by targeting multiple components of the RIG-I-MAVS, TLR3-TRIF, and cyclic GMP-AMP synthase (cGAS)-STING signaling pathways, all of which play essential roles in the immune response against viral infections (2, 3). ORF9b of both SARS-CoV and SARS-CoV-2 was also associated with the versatile adapter TOM70, resulting in immune evasion (4). Therefore, illustrating the molecular details of ORF9b interactions with host proteins may be beneficial for designing inhibitors against SARS-CoV-2 infection and regulating the host immune response.

Ubiquitination and deubiquitination are involved in regulating many biological processes and are widely exploited by diverse pathogens or hosts to antagonize each other. HIV-1 is a well-studied pathogen that manipulates the host's ubiquitin network to antagonize host restriction factors (5–9). In contrast, the host also utilizes the ubiquitin-proteasome system (UPS) to destabilize viral proteins such as deubiquitination by the deubiquitinating enzyme (DUB) USP21 of the HIV-1 protein Tat, which leads to Tat instability (10). Recent reports have shown that the E3 ubiquitin ligases RNF5 and CRL2 (ZYG11B) are recruited by membrane protein and ORF10 protein of SARS-CoV-2, respectively; However, CRL2 is dispensable for SARS-CoV-2 infection (11, 12). Guo et al reported that DUB USP13 is hijacked by SARS-CoV-2 nsp13 and prevents its degradation (13). PLpro encoded by SARS-CoV-2 played a DUB role in antagonizing the host immune response by removing ubiquitin-like ISG15 protein modification (14). However, it is largely unknown whether or how ubiquitination regulates SARS-CoV-2 infection and its viral proteins.

We hypothesize that the host may employ the UPS to target some viral proteins of SARS-CoV-2 for degradation, while viral proteins may hijack DUBs to prevent degradation. Therefore, we screened whether some viral proteins of SARS-CoV-2 were affected by UPS and found that ORF9b can be ubiquitinated at both K48 and K63 sites and degraded through the proteasomal pathway. Interestingly, USP29 can stabilize ORF9b expression by deubiquitinating ORF9b and blocking its degradation. Moreover, USP29 promoted the virulence of VSV-eGFP and transcription and replication-competent SARS-CoV-2 virus-like particles (trVLP) by enhancing ORF9b-mediated inhibition of IFN induction and NF-κB activation. Our study revealed a previously unrecognized interplay between the host DUB USP29 and SARS-CoV-2 ORF9b protein. Therefore, this study provides a new target for SARS-CoV-2 prevention and treatment.

## RESULTS

### ORF9b can be ubiquitinated and degraded through the proteasomal pathway.

**FIG 1:**
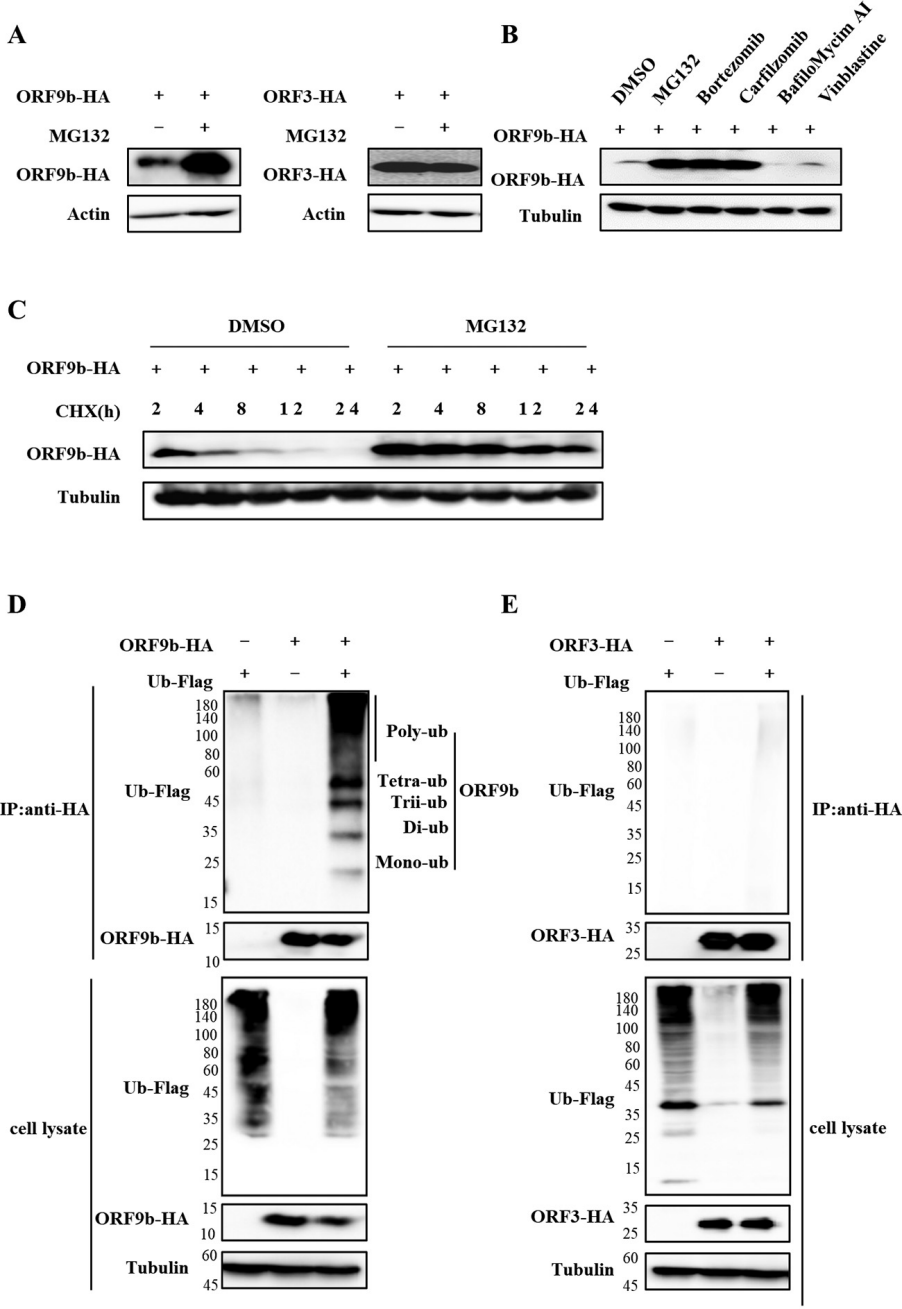
ORF9b is ubiquitinated and degraded through the proteasomal pathway. (A) Proteasomal inhibitor MG132 increased the expression of ORF9b but not ORF3a. (B) Proteasomal inhibitors, but not other inhibitors, increased ORF9b expression. The ORF9b-HA-tag expression vector was transfected into HEK293T cells, and then the cells were treated with 10 μM MG132, Bortezomib, Carfilzomib, BafiloMycim AI, Vinblastine, or DMSO for 12 h prior to harvest. The cell lysates were analyzed using immunoblot (IB). (C) MG132 stabilized ORF9b expression in cells treated with CHX. ORF9b-HA was transfected into HEK293T cells for 24 h, and the cells were treated with or without 10 μM MG132 for 10 h, then 50 μg/mL cycloheximide (CHX) was added. The cells were harvested at different time points, and their lysates were analyzed. ORF9b (D) but not ORF3 (E) are ubiquitinated. HEK293T cells transfected with ORF9b-HA or ORF3-HA plus Ub-Flag or the empty vector were treated with 10 mM MG132 for 12 h prior to harvest. Co-IP (with anti-HA) and IB analysis were performed.

To determine whether the ubiquitin-proteasomal pathway regulates ORF9b, we treated cells transfected with ORF9b with the proteasome inhibitor MG132 for 12 h prior to harvest. Then, the levels of the accessory proteins ORF3a and ORF7b, the nonstructural proteins nsp2, nsp3, nsp8, and nsp12, and the structural protein N as control were determined. We found that MG132 only increased ORF9b expression but did not affect ORF3a and other SARS-CoV-2 viral proteins (Fig. 1A and Fig; S1 in the supplemental material). Further investigation showed that the proteasome inhibitors bortezomib and carfilzomib increased the expression of ORF9b, but the lysosomal inhibitors BafiloMycim AI (Baf A1) and the autophagosome inhibitor vinblastine did not (Fig. 1B). Cycloheximide (CHX), a eukaryotic translational elongation and protein synthesis inhibitor, was used to treat the cells transfected with ORF9b. We found that CHX treatment caused rapid degradation of ORF9b in a time-dependent manner, whereas MG132 treatment stabilized the levels of ORF9b (Fig. 1C). These results suggested that ORF9b is regulated by ubiquitination and proteasomal degradation. Using a coimmunoprecipitation (co-IP) assay, we confirmed that ORF9b, but not ORF3a, was ubiquitinated (Fig. 1D and E).

10.1128/mbio.01300-22.1 FIG S1 Proteasomal inhibitor MG132 does not affect the expression of other SARS-CoV-2 viral proteins. (A–F) HEK293T cells were transfected with Flag-tagged SARS-CoV-2 viral protein expression vectors and treated with 10 mM MG132 for 12 h prior to harvest. Then cells were subjected to SDS-PAGE and IB analysis. Download FIG S1, TIF file, 0.3 MB. Copyright © 2022 Gao et al. 2022 Gao et al. https://creativecommons.org/licenses/by/4.0/ This content is distributed under the terms of the Creative Commons Attribution 4.0 International license.

### USP29 acts as a DUB preventing ORF9b degradation.

**FIG 2:**
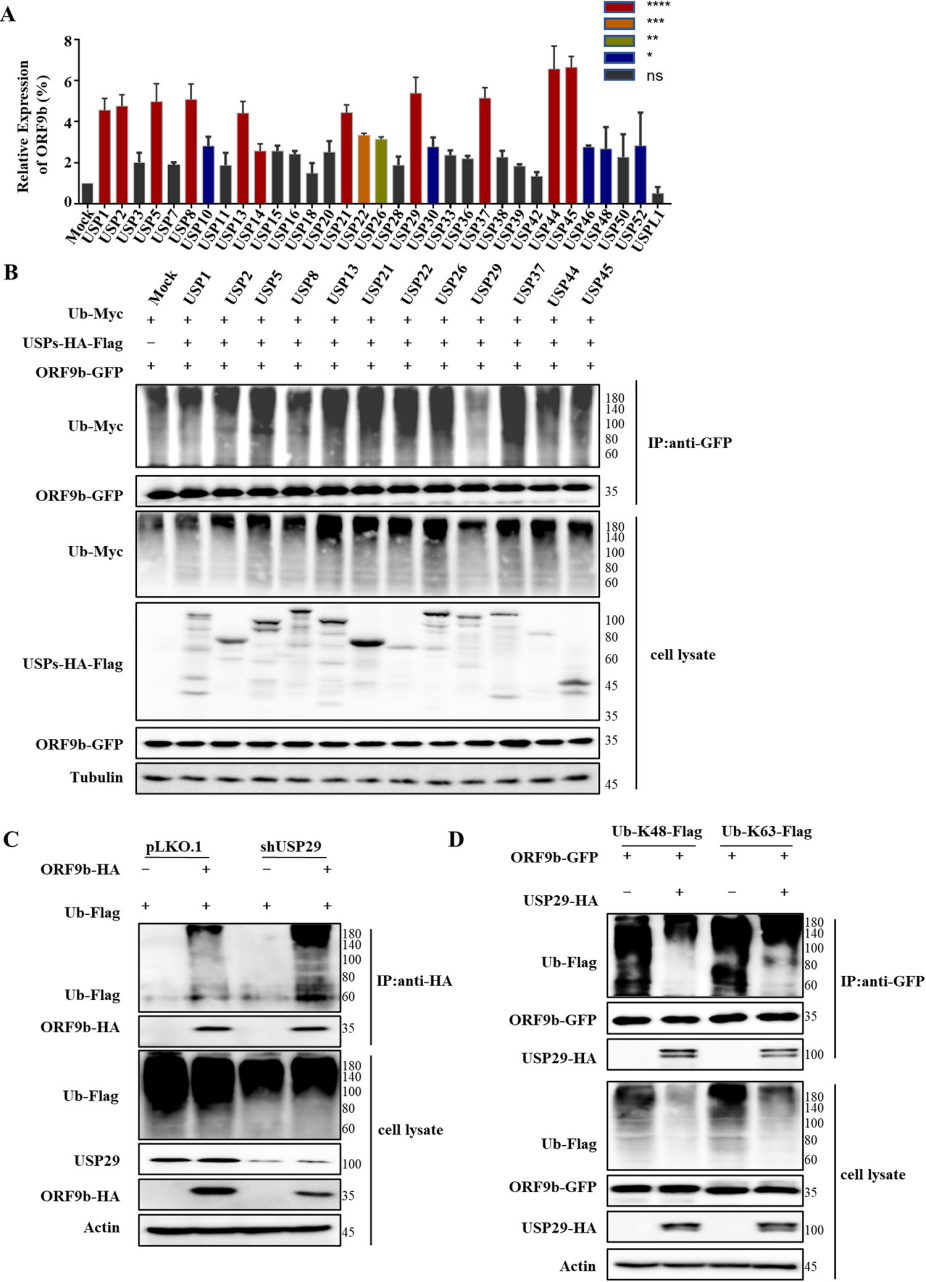
USP29 prevents ORF9b degradation by ubiquitination. (A) Effect of 33 deubiquitinase USPs on ORF9b expression. ORF9b-HA and USPs-HA-Flag or control vector were cotransfected into HEK293T cells. Cells were harvested at 48 h posttransfection and analyzed using IB. Quantification of ORF9b expression was performed using ImageJ2X, its expression in cells cotransfected with ORF9b, and the control vector was set to 100%. Statistical significance was analyzed using two-sided unpaired *t* tests (NS, not significant; *, *P < *0.05; **, *P < *0.01; ***, *P < *0.001; ****, *P < *0.0001). (B) USP29 deubiquitinated ORF9b. HEK293T cells were transfected with ORF9b-GFP, Ub-Myc, and USPs-HA-Flag, and treated with 10 mM MG132 for 12 h prior to harvest. Cell lysates were immunoprecipitated with anti-GFP antibodies conjugated to agarose beads. Cell lysates and precipitated samples were analyzed using IB with the corresponding antibodies. (C) USP29 silencing reduced deubiquitination of ORF9b. ORF9b ubiquitination in USP29 silencing and control cells were analyzed using Co-IP (with anti-Flag) and IB. (D) USP29 could cleave both K48- and K63-linked ubiquitin chains of ORF9b.

Ubiquitination can be reversed by DUBs, resulting in effects opposite to ubiquitination. Among them, the largest subfamily of USP has attracted considerable attention. We first screened the effect of some USPs on the expression of ORF9b and observed that several USPs, such as USP1, USP2, USP5, USP8, USP13, USP21, USP22, USP26, USP29, USP37, USP44, and UPS45, increased ORF9b expression by up to 4-fold, which were chosen for further investigation (Fig. 2A). A co-IP assay was employed to examine whether these USPs stabilized ORF9b expression by reversing ubiquitination and blocking its degradation. Only USP29 decreased the degree of ORF9b ubiquitination, whereas the other USPs had no effect, suggesting that USP29 stabilizes ORF9b expression via deubiquitination (Fig. 2B). Overexpression of USP29 resulted in results similar to those obtained following MG132 treatment in the CHX treatment assay (Fig. S2A–B). Accordingly, USP29 knockdown reduced deubiquitination of ORF9b, and the expression of ORF9b was decreased (Fig. 2C and S2C); the efficiency of USP29 knockdown was confirmed by immunoblotting and RT-qPCR assays (Fig. S2D–E). We further observed that ORF9b could be ubiquitinated at both K48 and K63 residues, whereas USP29 could reduce both K48- and K63-linked ubiquitination of ORF9b (Fig. 2D).

10.1128/mbio.01300-22.2 FIG S2 USP29 can increase ORF9b expression. (A) USP29 can significantly increase the expression of ORF9b, like MG132. (B) Overexpression of USP29 prolonged the half-life of ORF9b. HEK293T cells were transfected with ORF9b-HA, USP29-HA, or control vector and then treated with 50 μg/mL of CHX and harvested at the indicated times. (C) Knockdown of USP29 decreased the stability of ORF9b. USP29 was silenced, and control cells were transfected with ORF9b-HA. Forty-eight hours later, the cells were harvested and analyzed using IB. (D–E) Knockdown degree of USP29 in 293T cells. Protein levels (D) and mRNA levels (E) of USP29 were analyzed using IB and RT-PCR, respectively. Download FIG S2, TIF file, 0.4 MB. Copyright © 2022 Gao et al. 2022 Gao et al. https://creativecommons.org/licenses/by/4.0/ This content is distributed under the terms of the Creative Commons Attribution 4.0 International license.

### Deubiquitinase activity and the C-terminus of USP29 are required for ORF9b deubiquitination.

**FIG 3:**
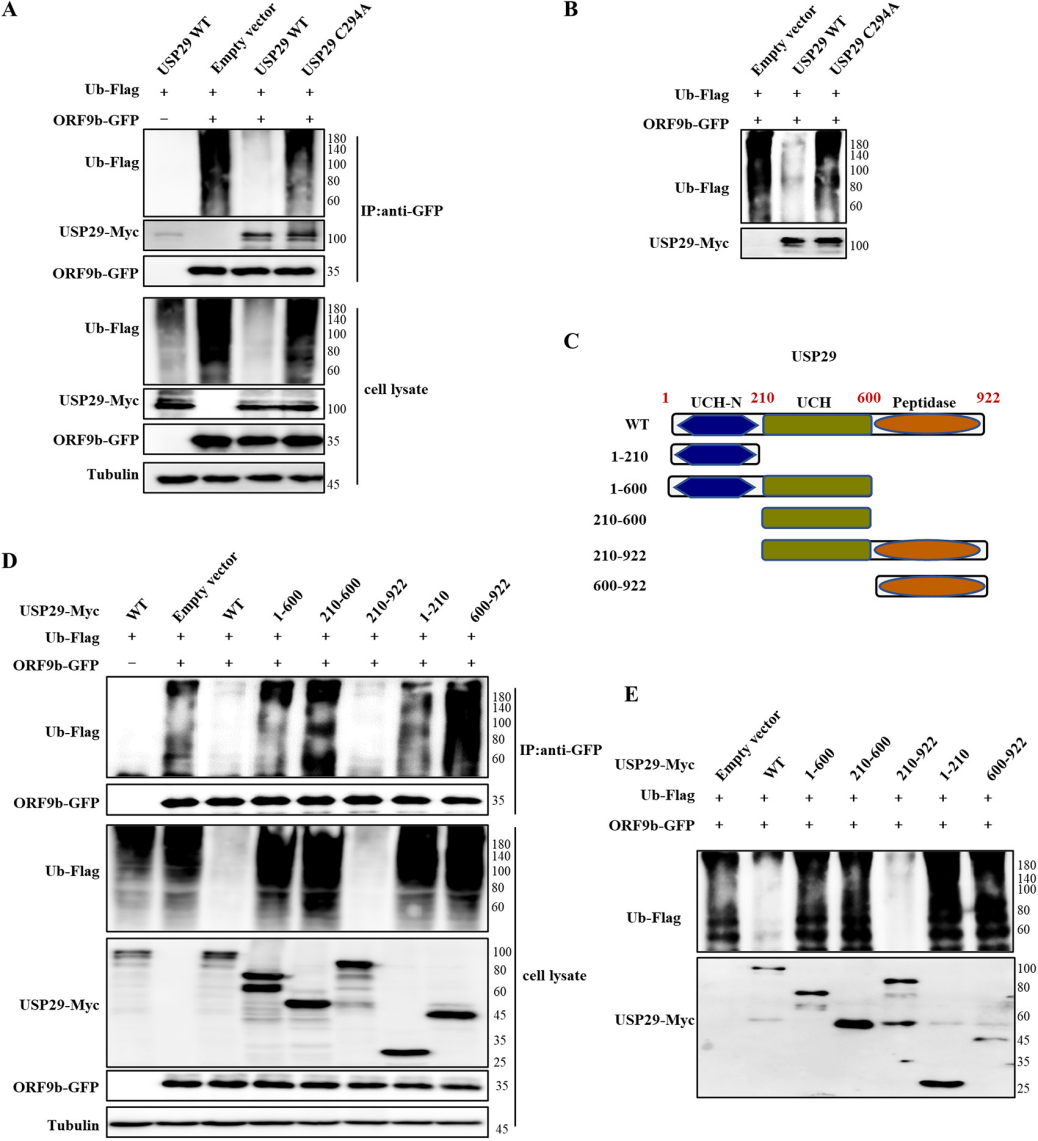
The enzymatic activity and the C-terminus of USP29 are required for ORF9b deubiquitination. (A) USP29 deubiquitinase-deficient C294A mutant could not deubiquitinate ORF9b *in vivo*. HEK293T cells transfected with the indicated expression vectors were treated with 10 mM MG132 for 12 h prior to harvest. Cell lysates were immunoprecipitated by protein G agarose beads conjugated with anti-GFP antibodies. Cell lysates and precipitated samples were analyzed using IB with the corresponding antibodies. (B) *In vitro* deubiquitination assay. Ubiquitinated ORF9b was immunoprecipitated from HEK293T cells transfected with ORF9b-GFP and Ub-Flag by anti-GFP antibody-conjugated protein G agarose beads. USP29 or its mutant was immunoprecipitated from HEK293T cells overexpressing USP29-Myc or its mutant using anti-Myc antibody-conjugated protein G agarose. Ubiquitinated ORF9b was incubated with purified USP29 or its mutant in deubiquitination buffer for 12 h at 37°C, and then was analyzed using IB. (C) The schematic represents USP29 WT and mutants. The C-terminus of USP29 is required for ORF9b deubiquitination *in vivo* (D) and *in vitro* (E) assays. The experimental process was similar to A and B.

To identify whether the deubiquitinase activity of USP29 is required for the deubiquitination and stabilization of ORF9b, we generated a USP29 deubiquitinase-deficient mutant (C294A). As expected, the USP29 C294A mutant lost the ability to deubiquitinate ORF9b (Fig. 3A). We further designed an *in vitro* deubiquitination assay by mixing ORF9b-GFP and USP29 WT or C294A mutant, immunoprecipitated from cotransfected ORF9b-GFP with ubiquitin-flag using anti-GFP beads, or from cells transfected with USP29-Myc wild type (WT) or C294A mutant using anti-Myc beads. The results also showed that USP29 WT, but not the C294A mutant, efficiently deubiquitinated ORF9b (Fig. 3B).

USP29 comprises a ubiquitin carboxyl-terminal hydrolase (UCH), UCH-N, and peptidase domains. To determine the functional domain of USP29 required for ORF9b deubiquitination, we generated several truncated mutants (Fig. 3C). The results showed that mutants 1-600, 210-600, 1-210, and 600-922 could not deubiquitinate ORF9b. Only mutant 210-922 maintained a similar deubiquitination ability as USP29 WT, suggesting that except for the UCH domain, the C-terminal peptidase domain (600-922 amino acid [aa]) of USP29 is also required for ORF9b deubiquitination (Fig. 3D). The *in vitro* deubiquitination assay yielded similar results (Fig. 3E). The interaction between USP29 and ORF9b is a prerequisite to exercise DUB activity. We performed forward and reverse IP assays, which showed the interaction between USP29 and ORF9b (Fig. S3A–B). Interestingly, we found that 1-210, 210-600, and 210-922 mutants showed a similar binding ability with ORF9b-like WT, and 1-600 mutant showed an even stronger binding ability compared to USP29 WT, whereas 600-922 mutant showed weaker binding with ORF9b (Fig. S3C). The phenomenon of stronger binding ability shown by USP29 1-600 mutant with ORF9b had been reported previously, and a little different is that USP29 210-600 mutant (UCH domain) showed stronger interaction with cGAS compared to USP29 WT (15). Altogether, the UCH-N and UCH domains of USP29 are necessary for ORF9b binding.

10.1128/mbio.01300-22.3 FIG S3 USP29 interacts with ORF9b. (A–B) USP29 interacts with ORF9b. (A) Immunoprecipitation (with anti-HA) and IB analysis of HEK293T cells transfected with plasmids encoding ORF9b-HA and USP29-Myc for 48 h. (B) Immunoprecipitation (with anti-Myc) and IB analysis (with anti-Myc or anti-HA) of HEK293T cells transfected with plasmids encoding ORF9b-HA and USP29-Myc for 48 h. (C) The N-terminus of USP29 is required for ORF9b interaction. HEK293T cells cotransfected with ORF9b-HA and USP29-Myc or mutants were subjected to Co-IP with anti-Myc antibody-conjugated agarose beads. The immunoprecipitates and lysates were analyzed using IB. Quantification of ORF9b interaction with USP29 was performed using ImageJ2X. Data were normalized to pulldown USP29 WT or mutations, respectively. The interaction of USP29 WT and ORF9b was set 1. Statistical significance was analyzed using two-sided unpaired *t* tests (NS, not significant; *, P < 0.05; ***, P < 0.001). Download FIG S3, TIF file, 0.6 MB. Copyright © 2022 Gao et al. 2022 Gao et al. https://creativecommons.org/licenses/by/4.0/ This content is distributed under the terms of the Creative Commons Attribution 4.0 International license.

### Functional domains and ubiquitination sites in ORF9b for USP29 binding and deubiquitination.

**FIG 4:**
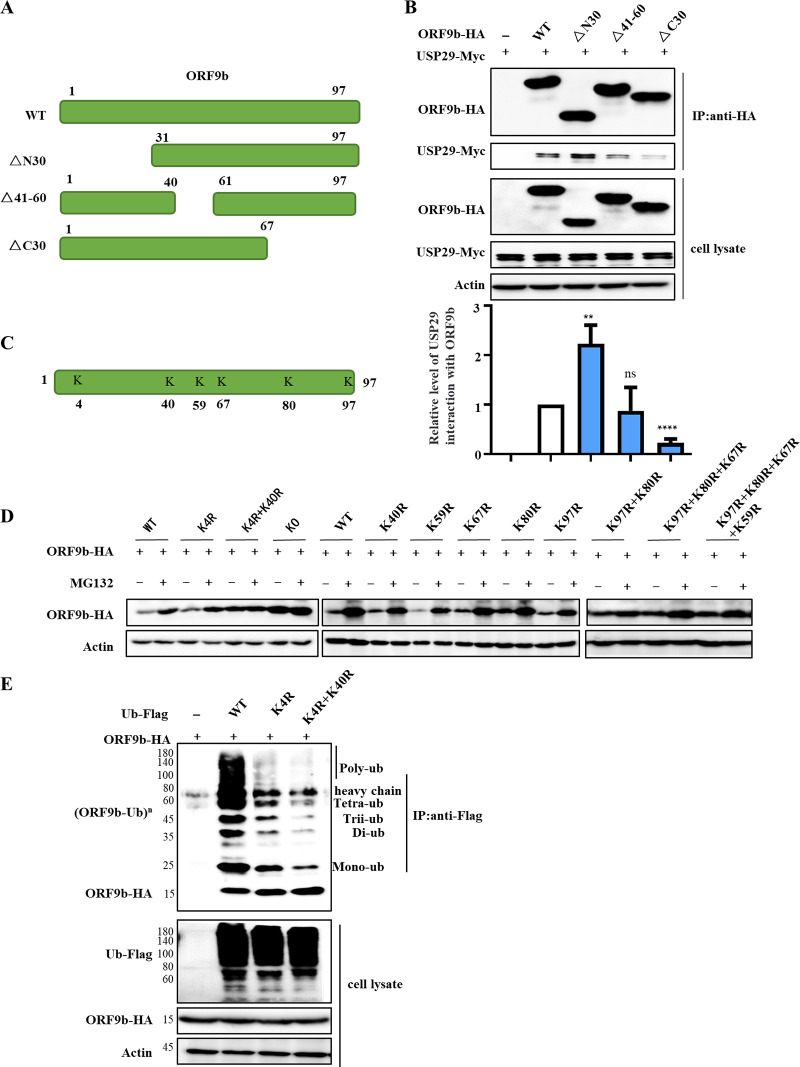
The ubiquitinated sites of ORF9b. (A) The schematic represents ORF9b WT and mutants used in the study. (B) The interaction between ORF9b and its mutants with USP29. HEK293T cells cotransfected with USP29-Myc and ORF9b-HA WT or mutants were subjected to Co-IP with anti-HA agarose beads. The immunoprecipitates and lysates were analyzed using IB. Quantification of USP29 interaction with ORF9b was performed using ImageJ2X. Data were normalized to pulldown ORF9b WT or mutations, respectively. The interaction of ORF9b WT and USP29 was set 1. Statistical significance was analyzed using two-sided unpaired *t* tests (NS, not significant; **, *P < *0.01; ****, *P < *0.0001). (C) Schema showing the lysines (K) of SARS-CoV-2 ORF9b. (D) The stability of ORF9b lysine mutants. (E) The ubiquitination of ORF9b lysine mutants. HEK293T cells transfected with ORF9b WT or mutants plus ubiquitin-Flag were treated with 10 mM MG132 for 12 h prior to harvest. Cell lysates were immunoprecipitated by protein G agarose beads conjugated with anti-Flag antibodies. Cell lysates and precipitated samples were analyzed using IB.

SARS-CoV-2 ORF9b contains two alpha helices (α1 and α2) and eight beta sheets (β1–β8). To map the ORF9b domain required for USP29 interaction and deubiquitination, we constructed truncated mutants, as described in a recent report (3) (Fig. 4A). IP assays showed that ORF9b ΔN30 and Δ41-60 mutants maintained the ability to interact with USP29, even stronger binding for ΔN30, whereas ΔC30 mutant showed weaker binding ability with USP29 compared to ORF9b WT, indicating that the C-terminus of ORF9b is required for USP29 binding (Fig. 4B). To determine the ubiquitination sites in ORF9b, we analyzed the full length of ORF9b and found six lysine sites, which were subsequently substituted by arginine separately or in combination (Fig. 4C). We first examined the stability of ORF9b mutants with or without MG132 and observed that only the K4R-K40R mutant was not degraded, which was similar to the result of the degradation of resistant KO (all lysines were mutated), indicating that the two lysines might be ubiquitinating sites (Fig. 4D). Consistent with our hypothesis, we observed that the single K4R mutant of ORF9b showed reduced ubiquitination, whereas a lower degree of ubiquitination was observed in the two-site mutant, K4R, and K40R (Fig. 4E).

### USP29 enhances ORF9b-mediated type I IFN and NF-κB inhibition.

**FIG 5:**
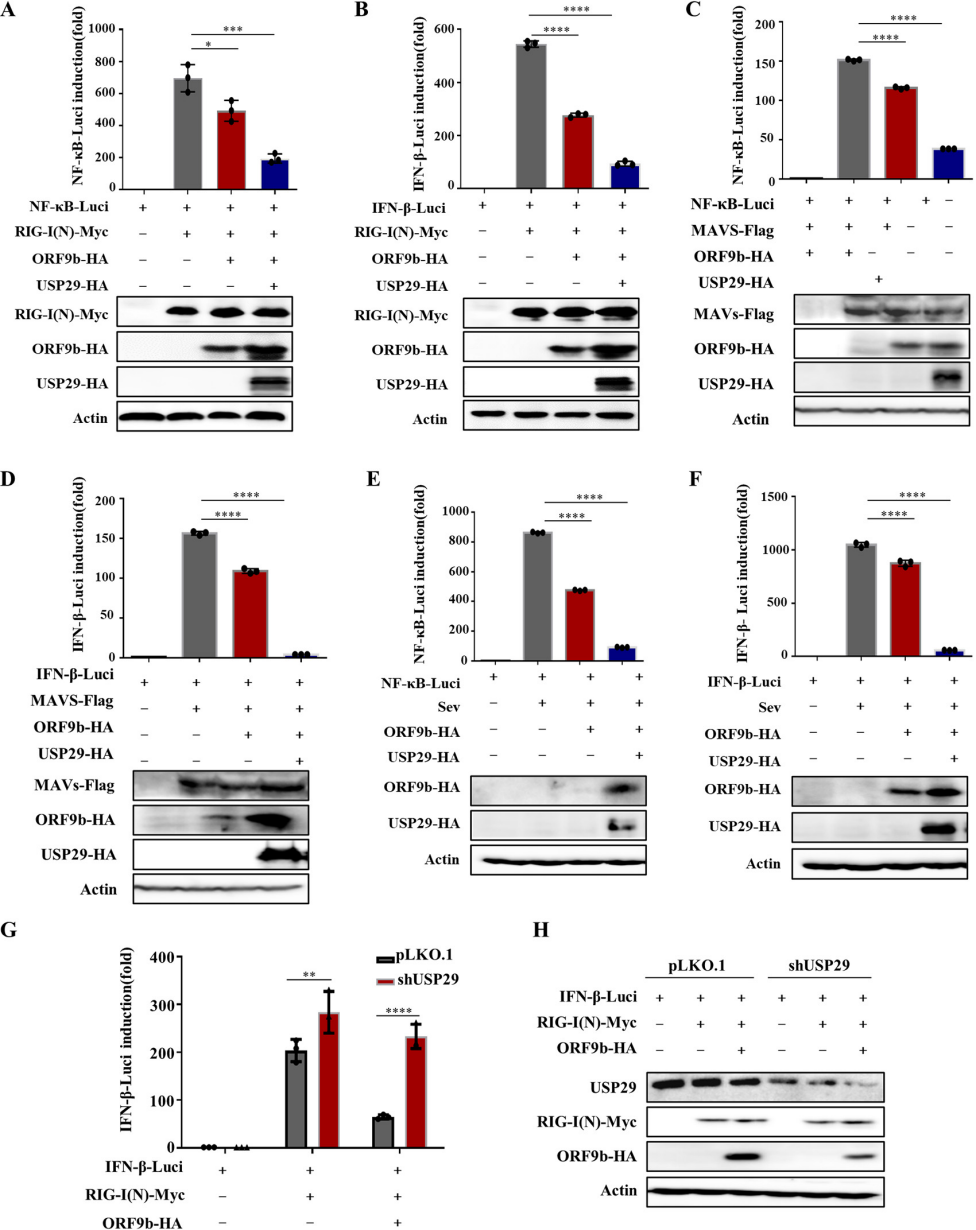
USP29 enhances ORF9b-mediated IFN and NF-κB inhibition by increasing ORF9b expression. (A–D) USP29 promotes ORF9b inhibition on RIG-N or MAVS to activate IFN-β/NF-κB promoters. HEK293T cells were transfected with an IFN-β/NF-κB promoter Luciferase reporter plasmid, renilla Luciferase reporter plasmid, or ORF9b-HA, along with a control plasmid or USP29-HA for 24 h. Then Luciferase reporter activities were induced by transfection of RIG-I(N)-Myc or MAVS-Flag expressing vectors into HEK293T cells for another 24 h. Cell lysate samples were analyzed using IB with the corresponding antibodies (lower panels). NF-κB-Luc (A, C) and IFN-β-Luc (B, D) reporter activities are normalized to renilla Luciferase and shown as fold induction. (E–F) USP29 promotes ORF9b inhibition of activation of IFN-β/NF-κB promoters by SeV infection. HEK293T cells were transfected with an IFN-β/NF-κB reporter plasmid, renilla reporter plasmid, or ORF9b-HA, along with a control plasmid or USP29-HA for 24 h. Then, Luciferase reporter activities were induced by SeV infection for 12 h. Cell lysate samples were analyzed using IB with the corresponding antibodies (lower panels). NF-κB-Luc (E) and IFN-β-Luc (F) reporter activities are normalized to that of renilla Luciferase and shown as fold induction. (G–H) Knockdown of USP29 reduced ORF9b-mediated IFN inhibition. USP29 silencing or control HEK293T cells were transfected with IFN-β reporter plasmid, renilla reporter plasmid, ORF9b-HA, control plasmid, or USP29-HA for 24 h. Then Luciferase reporter activities were induced by transfection of RIG-I(N)-Myc into USP29 silenced or control HEK293T cells for 24 h. (G) IFN-β-Luc reporter activities are normalized to renilla Luciferase and shown as fold induction. (H) Cell lysate samples were analyzed using IB with the corresponding antibodies. Data are represented as means ± SDs calculated from three independent experiments (****, *P < *0.0001).

Recent studie reported that ORF9b immediately accumulates during SARS-CoV-2 infection and inhibits the activation of IFN-β and NF-κB promoter following expression of RIG-I(N) (the constitutively active form of RIG-I) (3). Based on these data, we next examined the effect of USP29 on ORF9b-mediated IFN-β inhibition. As expected, overexpression of USP29 increased the expression of ORF9b, resulting in stronger IFN-β and NF-κB inhibition than ORF9b alone (Fig. 5A and B). Even at the higher dose, the USP29 C294A mutant did not affect ORF9b-mediated inhibition of IFN-β induction and NF-κB activation (Fig. S4A-B). Accordingly, USP29 WT but not C294A increased the expression of ORF9b (Fig. S4A-B). In addition, USP29 enhanced ORF9b inhibition of IFN-β and NF-κB activation in response to various stimuli, such as MAVS and Sendai virus (SeV) (Fig. 5C–F). In contrast, knockdown of USP29 reduced the expression of ORF9b, thereby releasing IFN-β induction and NF-κB activation (Fig. 5G–H). Furthermore, we determined the mRNA levels of IFN-β and IFN-inducible genes ISG15 and OAS2 when ORF9b or ORF9b and USP29 were cotransfected with RIG-I(N) into HEK293T cells, as indicated. Similarly, USP29 enhanced ORF9b expression, further downregulating the mRNA levels of IFN-β, ISG15, and OAS2 compared with ORF9b alone, whereas USP29 alone showed no significant difference effect (Fig. S5A–D).

10.1128/mbio.01300-22.4 FIG S4 USP29 deubiquitinase activity is required to inhibit IFN and NF-κB activation. (A–B) HEK293T cells were transfected with IFN-β/NF-κB reporter plasmid, renilla reporter plasmid, ORF9b-HA, control plasmid, USP29-Myc, or its mutants (C294A) for 24 h. Then Luciferase reporter activities were induced by transfection of RIG-I(N)-Myc into HEK293T cells for 24 h. NF-κB-Luc (A) and IFN-β-Luc (B) reporter activities are normalized to that of renilla Luciferase and shown as fold induction. Cell lysate samples were analyzed using IB with the corresponding antibodies (lower panels). Statistical significance was analyzed using two-sided unpaired *t* tests (NS, not significant; ****, P < 0.0001). Download FIG S4, TIF file, 0.5 MB. Copyright © 2022 Gao et al. 2022 Gao et al. https://creativecommons.org/licenses/by/4.0/ This content is distributed under the terms of the Creative Commons Attribution 4.0 International license.

10.1128/mbio.01300-22.5 FIG S5 USP29 promotes ORF9b inhibition of the antiviral IFN response. (A–D) HEK293T cells were transfected with ORF9b-HA and empty vector or USP29-HA expressing plasmids for 24 h. Then cells were transfected with RIG-I(N)-Myc for another 24 h. RT-qPCR was conducted to determine the mRNA expression levels of IFN-β (A), ISG15 (B), and OAS2 (C). (D) Protein samples in Fig. S5A-C were analyzed using IB. (E–F) Caco2-N^int^ cells were transfected with ORF9b-HA and empty vector or USP29-HA expressing plasmids for 24 h. Then cells were infected with SARS-CoV-2 trVLPs at an MOI of 1 for 24 h. RT-qPCR was conducted to determine the mRNA levels of IFN-β (E) and ISG15 (F). Download FIG S5, TIF file, 0.5 MB. Copyright © 2022 Gao et al. 2022 Gao et al. https://creativecommons.org/licenses/by/4.0/ This content is distributed under the terms of the Creative Commons Attribution 4.0 International license.

### USP29 increases the virulence of VSV-eGFP and SARS-CoV-2 trVLP.

**FIG 6:**
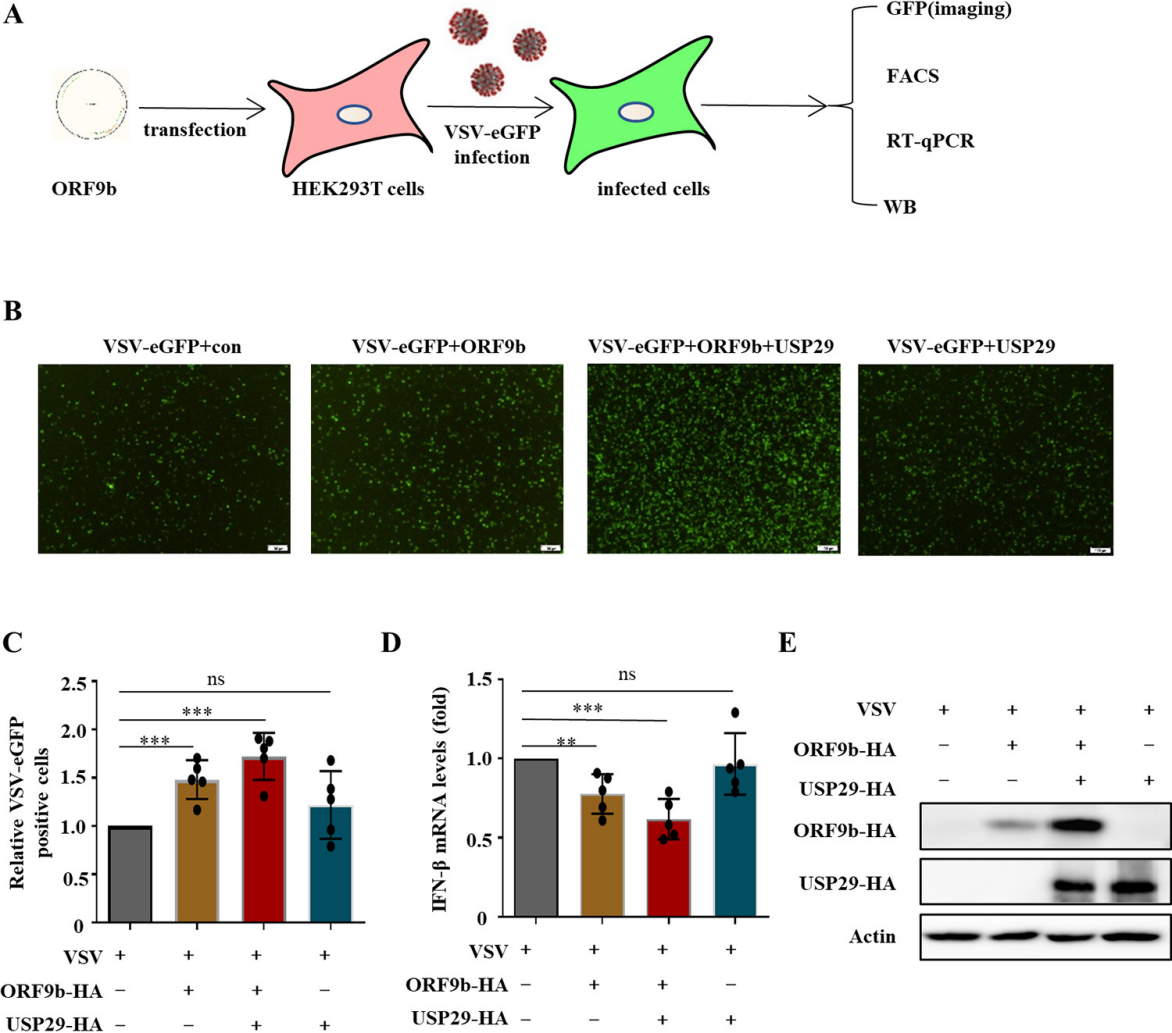
USP29 increases VSV viral infectivity through stabilizing ORF9b. (A) Schematic presentation of assessment of USP29 increases VSV viral infectivity through stabilizing ORF9b. (B–E) At 24 h posttransfection of empty vector or ORF9b and USP29-HA expressing plasmids, HEK293T cells were infected with VSV-eGFP for 12 h. (B) Fluorescent images were taken to examine VSV-eGFP proliferation. (C) eGFP positive cells were analyzed using flow cytometry, and the mRNA expression of IFN-β was determined by RT-qPCR (D). (E) Cell lysate samples were analyzed using IB with the corresponding antibodies.

**FIG 7:**
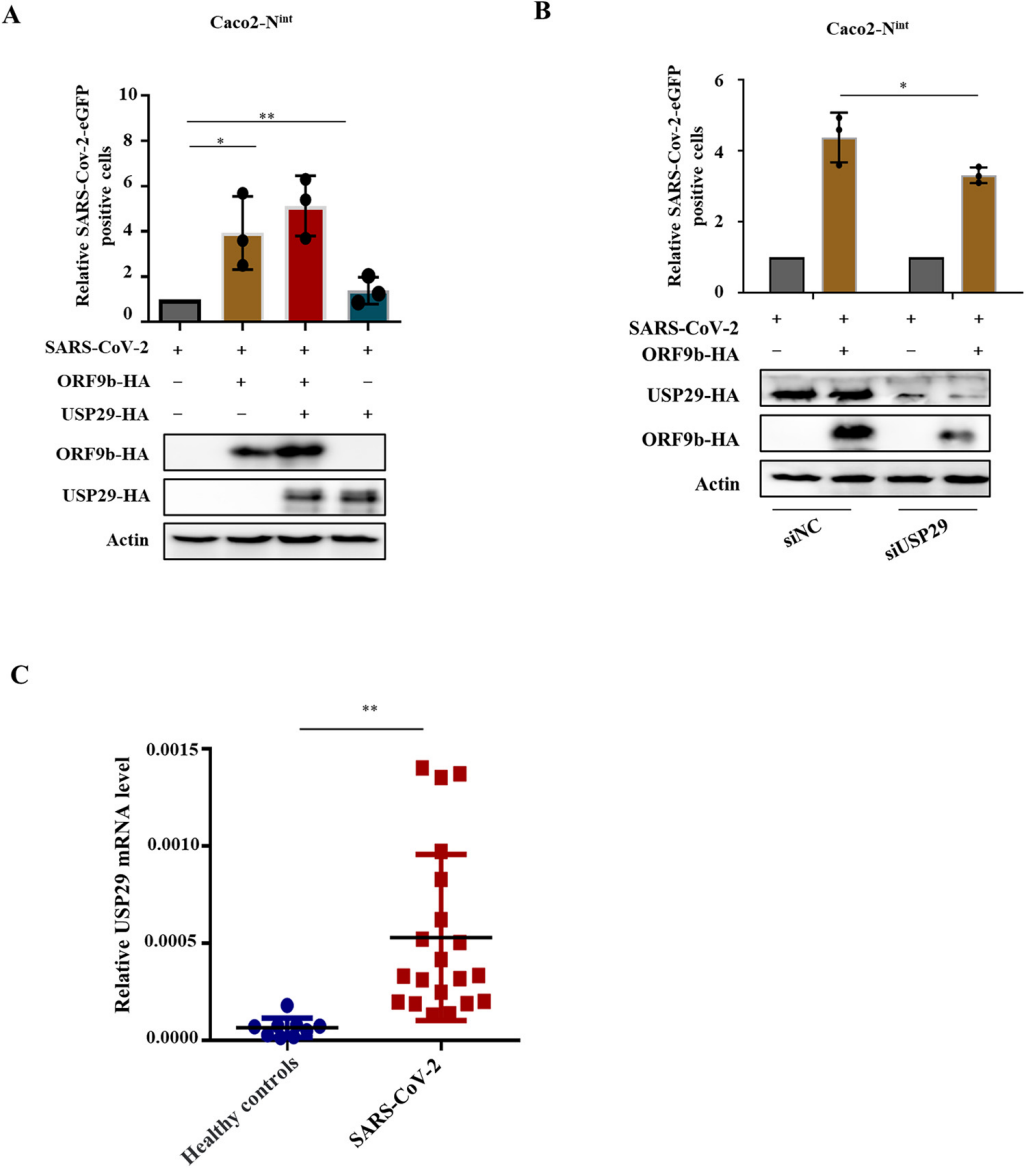
USP29 increases SARS-CoV-2 trVLP infectivity through stabilizing ORF9b. (A) Caco-2-N^int^ cells were transfected with ORF9b-HA plus control plasmid or USP29-HA for 24 h. Then cells were infected with SARS-CoV-2 trVLPs at a multiplicity of infection (MOI) of 0.1 for 48 h. The infection was analyzed using flow cytometry to detect eGFP-positive cells (top). Protein expression was analyzed using IB (bottom). (B) USP29 silencing reduced SARS-CoV-2 trVLPs infectivity by reducing ORF9b expression. Caco-2-N^int^ cells were transfected with ORF9b-HA for 24 h. Infection and IB were conducted as described in A. (C) Total mRNA was extracted from PBMC cells of a healthy person or recovered SARS-CoV-2 patients. USP29 mRNA levels were determined using RT-qPCR. Statistical significance was analyzed using two-sided unpaired *t* tests (NS, not significant; *, *P < *0.05; **, *P < *0.01; ***, *P < *0.001; ****, *P < *0.0001).

To examine whether USP29-mediated stabilization of ORF9b affects viral infection, we first examined the effect of USP29 on VSV-eGFP replication in the presence of ORF9b. Green fluorescence assay showed that ORF9b moderately increased VSV-eGFP replication, whereas USP29 overexpression further promoted ORF9b enhancement in VSV-eGFP replication. We also observed that USP29 alone did not affect VSV-eGFP replication (Fig. 6A to C), indicating that USP29 affects viral replication in an ORF9b-dependent manner. Accordingly, USP29 enhanced ORF9b-mediated IFN-β inhibition, while USP29 alone did not affect IFN-β mRNA levels (Fig. 6D). As expected, USP29 increased ORF9b expression (Fig. 6E). To further validate the role of USP29 in SARS-CoV-2 infection, we employed a biosafety level-2 cell culture system for the production of SARS-CoV-2 trVLPs (16). The results showed that overexpression of ORF9b increased the SARS-CoV-2 trVLP infectivity, whereas USP29 further enhanced the SARS-CoV-2 trVLP infectivity by upregulating the expression of ORF9b (Fig. 7A). In USP29-silencing Caco2-N^int^ cells, the SARS-CoV-2 trVLP infectivity was reduced due to reduced ORF9b expression than in siNC cells (Fig. 7B, Lanes 1 and 3). Accordingly, ORF9b overexpression reduced SARS-CoV-2-induced mRNA levels of IFN-β and ISG15, whereas USP29 further enhanced ORF9b-mediated downregulation of IFN-β and ISG15 mRNA levels (Fig. S5E-F). To investigate whether USP29 expression is correlated with COVID-19 disease, we examined the mRNA levels of USP29 in healthy people and SARS-CoV-2 infected patients and observed that the mRNA level of USP29 in patients was higher than in healthy people, suggesting that the expression level of USP29 in the peripheral blood mononuclear cells (PBMCs) of SARS-CoV-2 infected individuals is closely associated with COVID-19 disease progression (Fig. 7C).

## DISCUSSION

Ubiquitination and deubiquitination are involved in many cellular processes and have emerged as novel targets for developing therapeutic agents. The UPS is the primary cytosolic proteolytic machinery for the degradation of various proteins, including viral proteins, whereas viral proteins manipulate host proteins to reverse this process; for instance, the first identified DUB USP7 is associated with viral infection (17, 18). USP29 has recently been reported to play an important role in cancer metabolism, progression, and prognosis (19–22). USP29 cooperates with phosphatase SCP1 to stabilize Snail protein, thereby promoting gastric cancer cell migration and mediating HIFα stabilization to induce sorafenib resistance in hepatocellular carcinoma cells by upregulating glycolysis (4). USP29 also regulates innate immune responses and autoimmunity by stabilizing cGAS (15). However, the direct regulation of USP29 on viral proteins or viral infections has not yet been reported.

SARS-CoV-2 has been a major threat to humankind due to its high morbidity and elevated mortality rate, especially the constantly emerging variants of SARS-CoV-2 like Omicron. Therefore, the underlying mechanism of SARS-CoV-2 pathogenesis needs to be investigated in detail to develop drugs and vaccines. In particular, it is necessary to discover the regulatory function of ubiquitination and deubiquitination of viral proteins in SARS-CoV-2 infection. SARS-CoV-2 ORF7a was recently ubiquitinated at K63, enhancing ORF7a-mediated IFN-I inhibition (23). Only nsp13 was found to be regulated by USP13 in a ubiquitin-proteasome-dependent manner (13). In our study, we found for the first time that ORF9b can be degraded by the proteasomal degradation pathway (Fig. 1 and 2; Fig. S1). We could not identify which E3 ligase induces its degradation, which is worth investigating in the future. The K48- and K63-linked chains were the two most abundant linkage types. The K48-linked chain targets proteins for proteasomal degradation, whereas the K63-linked polyubiquitin chain regulates proteasome-independent events, such as immune responses.

Interestingly, a recent study reported that K63 ubiquitination plays a critical role in proteasomal degradation by serving as a “seed” for K48/K63 branched linkages preferentially associated with proteasomes in cells (24). Here, we observed that ORF9b could be ubiquitinated at both K48 and K63 residues, while USP29 can deconjugate both K48- and K63-linked chains (Fig. 2D), which is consistent with the fact that most USPs are nonspecific in cleaving ubiquitin chains 25). The deubiquitination capacity of DUBs is dependent or independent of their catalytic activity (25). Here, we demonstrated that the deubiquitinating enzymatic activity of USP29 is responsible for ORF9b stability. Moreover, the C-terminal 600-922 aa of USP29 is also required for its deubiquitination activity (Fig. 3). To verify whether USP29 is an effective target for SARS-CoV-2 therapy, we examined the possible correlation between ORF9b expression and COVID-19 disease progression. According to case statistics, we found higher USP29 mRNA levels in SARS-CoV-2-infected patients than in healthy people (Fig. 7C), indicating that SARS-CoV-2 might manipulate the host proteins to facilitate its propagation.

In summary, USP29 deubiquitinates SARS-CoV-2 ORF9b and prevents its degradation from the UPS, resulting in its higher expression at the protein level, which facilitates its degradation SARS-CoV-2 pathogenesis. Therefore, USP29 may be an important target for drug development and novel therapeutic strategies against SARS-CoV-2 infection.

## MATERIALS AND METHODS

### Plasmid construction.

The DNAs encoding for ORF9b-Flag/GFP, ORF3-Flag, ORF7b-Flag, NSP2/3/8/12-Flag, and N-Flag were synthesized by Shanghai Generay Biotech Co., Ltd. (Shanghai, China). ORF9b-HA was constructed with an N-terminal HA tag and was inserted between the SalI and BamHI sites of VR1012. ORF9b-HA△N30, △41-60, △C30, K4R, K40R, K59R, K67R, K80R, and K97R mutants were generated from ORF9b-HA by site-directed mutagenesis. All USPs with HA-Flag tags were purchased from Addgene (Watertown, MA, USA). USP29-HA/Myc and truncated mutants were constructed with a C-terminal HA or Myc tag and inserted between the SalI and BamHI sites of VR1012. USP29-HA C294A was generated from USP29-HA using site-directed mutagenesis. Ub-Flag/Myc (10), RIG-I(N)-Myc (26), MAVS, IFN-β-Luc, NF-κB-Luc, and Renilla (27) have been previously described. The SARS-CoV-2 GFP/ΔN (Wuhan-Hu-1, MN908947, Clade:19A) expression plasmid was constructed by replacing the regions encoding viral N (from nucleotide positions 28274 to 29533) based on the MN908947 genome with the GFP reporter gene. This process was performed as previously described (16). All the constructs were confirmed by DNA sequencing.

### Cell culture.

HEK293T cells (ATCC; CRL-11268) were obtained from American Type Culture Collection (ATCC; Manassas, VA, USA). Caco-2-N^int^ cells (Caco-2 cells stably expressing the SARS-CoV-2 N gene via lentiviral transduction) have been described in detail (16). HEK293T and Caco-2-N^int^ cells were maintained in Dulbecco's modified Eagle medium (DMEM; HyClone, Logan, UT, USA) supplemented with 10% (vol/vol) fetal bovine serum (FBS; 04-001-1; Biological Industries, Beit-Haemek, Israel) and 100 μg/mL penicillin/streptomycin in a humidified 5% (vol/vol) CO_2_ incubator at 37°C. All cell lines tested negative for Mycoplasma.

### Virus infection.

For VSV-eGFP infection, HEK293T cells were seeded in 12-well plates at a density of 1 × 10^5^ cells/well and transfected with the corresponding plasmids. Twenty-four h later, the cells were infected with VSV-eGFP for 12 h and collected for GFP fluorescence analysis by flow cytometry. For SARS-CoV-2 infection, we used a biosafety level-2 (BSL-2) cell culture system for the production of transcription and replication-competent SARS-CoV-2 virus-like-particles (Wuhan-Hu-1, MN908947, Clade:19A), which has been previously described in detail (16). The assay was approved by the Institutional Biosafety Committee of Jilin University. Caco-2-N^int^ cells were infected with trVLP at an MOI of 0.1 for 2 h, washed three times with PBS, and incubated in 2% FBS culture medium for 48 h. Infection was analyzed by flow cytometry to detect eGFP expression.

### Immunoblot analysis.

For immunoblot (IB) analysis of cell-associated proteins, expression plasmids were transfected into HEK293T or Caco2 N^int^ cells. At 48 h posttransfection, cells were harvested by centrifuge (1,000 *g*, 25°C for 5 min), lysed in lysis buffer (50 mM Tris–HCl, pH 7.8, 150 mM NaCl, 1% NP-40, 1% sodium deoxycholate, and 4 mM EDTA) and boiled in 1× loading buffer (0.08 M Tris [pH 6.8], 2.0% SDS, 10% glycerol, 0.1 M dithiothreitol, and 0.2% bromophenol blue) for 30 min at 100°C with occasional vortexing to shear cellular DNA. Cell lysates were subjected to SDS–PAGE. Proteins were transferred to polyvinylidene fluoride (PVDF) membranes and reacted with the appropriate antibodies, as described in the text. Membranes were then incubated with HRP-conjugated secondary antibodies, and protein bands were visualized using an ultrasensitive ECL Chemiluminescence Detection Kit (B500024; Proteintech, Rosemont, IL, USA).

### Co-immunoprecipitation (Co-IP).

HEK293T cells were then transfected with the corresponding plasmids. The cells were harvested by centrifugation (1,000 *g*, 25°C for 5 min), lysed in lysis buffer (50 mM Tris at pH 7.5, 150 mM NaCl, 1% NP-40, and complete protease inhibitor cocktail tablets) at 4°C for 3 h, and then centrifuged at 12,000 *g* for 10 min. Precleared cell lysates were mixed with antibody-conjugated protein G agarose beads and incubated overnight at 4°C. The next day, the beads were washed six times with washing buffer (20 mM Tris, pH 7.5, 100 mM NaCl, 0.1 mM EDTA, and 0.05% Tween 20) at 4°C, centrifuged at 800 × *g* for 1 min. The proteins were eluted with elution buffer (0.1 M glycine-HCl, pH 2.5) and analyzed by SDS-PAGE and immunoblotting.

### Generation of knockdown cell lines.

USP29-specific shRNA with the following target sites (5′-CCCATCAAGTTTAGAGGAT-3′) was cloned into the lentiviral vector pLKO.1-puro (Addgene). HEK293T cells were cotransfected with sh-USP29-pLKO.1 or pLKO.1 plus pRSV-Rev, pMDLg/pRRE, or pCMV-VSVG expression vectors using Lipofectamine 2000 (Invitrogen). At 48 h posttransfection, supernatants containing packaged lentiviruses were harvested and used to infect 293T cells for 48 h. Next, puromycin (5 μg/mL for HEK293T) was added to the culture to screen for stable cell lines.

### Dual-Luciferase reporter assays.

HEK293T cells were seeded in 12-well cell culture plates and transfected, using Lipofectamine 2000, with the corresponding expression plasmid or empty vector plus Luciferase reporter plasmid and 1 ng renilla Luciferase plasmid. At 24 h posttransfection, cells were transfected with plasmids encoding various RIG-I-MAVS signaling components for another 24 h or infected with SeV for another 12 h. Luciferase activity was measured using the Dual-Luciferase Reporter Assay System (E1910; Promega, Madison, WI, USA) according to the manufacturer's protocol using a GloMax 20/20 Luminometer (Promega).

### RNA extraction and quantitative real-time RT-qPCR.

According to the manufacturer's instructions, RNA was isolated from various cells using TRIzol reagent (15596-026; Invitrogen, Carlsbad, CA, USA). According to the manufacturer's instructions, RNA reverse transcription was performed using the EasyScript First-Strand cDNA Synthesis SuperMix (AE301; TransGen Biotech, Beijing, China). The cDNA was stored at −80°C until use. Quantitative real-time PCR (RT-qPCR) was performed on an Mx3005P instrument (Agilent Technologies, Stratagene, La Jolla, CA, USA) using Power SYBR Green PCR Master Mix (2×) (4367659; ABI, Carlsbad, CA, USA). RT-qPCR amplification of the target fragment was carried out as follows: initial denaturation at 95°C for 2 min, followed by 45 cycles at 95°C for 15 s, 57°C for 15 s, and 68°C for 20 s. Data were normalized to the housekeeping GAPDH gene, and the relative abundance of the target gene was calculated using Ct models. Primers used in this study are listed in Table 1.

**TABLE 1:** The sequence of primers for gene cloning and qPCR

Gene ID / name	Sequence (5′ to 3′)	Purpose
HA-ORF9b-△C30-For	ACGACGTCCCAGATTACGCGATGGGGCGCGATCAAAACAACGTCGGCC	Protein expression
HA-ORF9b-△C30-Rev	CGTTGTTTTGATCGCGCCCCATCGCGTAATCTGGGACGTCGTAAGGG	
HA-ORF9b-△41-60-For	AACAACGTCGGCCCCAATTCCCTCGAGGACAAGGCG	
HA-ORF9b-△41-60-Rev	TGTCCTCGAGGGAATTGGGGCCGACGTTGTTTTGATCG	
HA-ORF9b-△N30-For	TTCCCTCGAGGACAAGTACCCTTACGACGTCCCAGATTAC	
HA-ORF9b-△N30-Rev	CTGGGACGTCGTAAGGGTACTTGTCCTCGAGGGAATTTAAGG	
HA-USP29-For	CGTCGTCGACACGTGTGATCAGATATGATATCTCTAAAGGTATGT	
HA-USP29-Rev	CGCCTGGTCTAGAGCGGCCGCGATTTACGCGTAATCTGGGACGTCGTAAGGGTATTCCCCCTGAGGGATCACCCCTGC	
Myc-USP29-For	CGTCGTCGACACGTGTGATCAGATATGATATCTCTAAAGGTATGT	
Myc-USP29-Rev	CTGGTCTAGAGCGGCCGCGATTTAAAGATCTTCTTCTGATATGAGTTTTTGTTCTTCCCCCTGAGGGATCAC	
Myc-USP29 1-210-For	CGTCGTCGACACGTGTGATCAGATATGATATCTCTAAAGGTATGT	
Myc-USP29 1-210-Rev	GGTCTAGAGCGGCCGCGATTTAAAGATCTTCTTCTGATATGAGTTTTTGTTCTGGGTTCTTCCTATTGCTTTG	
Myc-USP29 1-600-For	CGTCGTCGACACGTGTGATCAGATATGATATCTCTAAAGGTATGT	
Myc-USP29 1-600-Rev	CTGGTCTAGAGCGGCCGCGATTTAAAGATCTTCTTCTGATATGAGTTTTTGTTCATTCTTGTCTGGTTCAACGG	
Myc-USP29 210-600-For	CGTCGTCGACACGTGTGATCAGATATGTCAAGTTTAGAGGATTTAG	
Myc-USP29 210-600-Rev	CTGGTCTAGAGCGGCCGCGATTTAAAGATCTTCTTCTGATATGAGTTTTTGTTCATTCTTGTCTGGTTCAACGG	
Myc-USP29 210-922-For	CGTCGTCGACACGTGTGATCAGATATGTCAAGTTTAGAGGATTTAG	
Myc-USP29 210-922-Rev	CTGGTCTAGAGCGGCCGCGATTTAAAGATCTTCTTCTGATATGAGTTTTTGTTCTTCCCCCTGAGGGATCAC	
Myc-USP29 600-922-For	GTCGTCGACACGTGTGATCAGATATGAATGCCGACCTACAAAGATTCC	
Myc-USP29 600-922-Rev	CTGGTCTAGAGCGGCCGCGATTTAAAGATCTTCTTCTGATATGAGTTTTTGTTCTTCCCCCTGAGGGATCAC	
Myc-USP29 C294A-For	GTTCCCCAATTTGGGAAACACCGCTTACATGAATGCAGTTTTACAATCGC	
Myc-USP29 C294A-Rev	GTAAAACTGCATTCATGTAAGCGGTGTTTCCCAAATTGGGGAACCCCTGCTG	
HA-ORF9b-K4R-For	CCAGATTACGCGATGGACCCCAGAATCAGCGAAATGCACCCCGCATTACG	
HA-ORF9b-K4R-Rev	GCGGGGTGCATTTCGCTGATTCTGGGGTCCATCGCGTAATCTGGGACGTC	
HA-ORF9b-K40R-For	CGATCAAAACAACGTCGGCCCCAGGGTTTACCCAATAATACTGCGTCTTGGTTC	
HA-ORF9b-K40R-Rev	AGACGCAGTATTATTGGGTAAACCCTGGGGCCGACGTTGTTTTGATCGCGCCC	
HA-ORF9b-K59R-For	CCGCTCTCACTCAACATGGCAAGGAGGACCTTAAATTCCCTCGAGGAC	
HA-ORF9b-K59R-Rev	AGGGAATTTAAGGTCCTCCTTGCCATGTTGAGTGAGAGCGGTGAACC	
HA-ORF9b-K67R-For	CCTTAAATTCCCTCGAGGACAGGGCGTTCCAATTAACACCAATAGC	
HA-ORF9b-K67R-Rev	GGTGTTAATTGGAACGCCCTGTCCTCGAGGGAATTTAAGGTCTTCC	
HA-ORF9b-K80R-For	CAATAGCAGTCCAGATGACCAGATTGGCTACTACCGAAGAGCTACC	
HA-ORF9b-K80R-Rev	CTTCGGTAGTAGCCAATCTGGTCATCTGGACTGCTATTGGTG	
HA-ORF9b-K97R-For	AATTCGTGGTGGTGACGGTAAGATACCCTTACGACGTCCCAGATTAC	
HA-ORF9b-K97R-Rev	CTGGGACGTCGTAAGGGTATCTTACCGTCACCACCACGAATTCGTCTG	
ShUSP29	CCCATCAAGTTTAGAGGAT	
SiUSP29	CCCAUCAAGUUUAGAGGAU	
USP29-For	CCAGGTGCCTCTTGACTCTC	qPCR
USP29-Rev	GCAGCTGGGTCAAGGTCATA	
GAPDH-For	GCAAATTCCATGGCACCGT	
GAPDH-Rev	TCGCCCCACTTGATTTTGG	
IFNβ-For	AAACTCATGAGCAGTCTGCA	
IFNβ-Rev	AGGAGATCTTCAGTTTCGGAGG	
IFNγ-For	GCAGGTCATTCAGATGTAGC	
IFNγ-Rev	TGGCTCTGCATTATTTTTCTG	
OSA2-For	AGTCTTAAGAGGCAACTCCG	
OSA2-Rev	AAGGGACTTCTGGATCTCG	
ISG15-For	CGCAGATCACCCAGAAGATCG	
ISG15-Rev	TTCGTCGCATTTGTCCACCA	

### Chemical synthesis of siRNA.

Chemically synthesized short interfering RNA (siRNA) and a nonspecific control were purchased from RiboBio Co. Ltd. (Guangzhou, China) to knock down USP29. The siUSP29 sequences were the same as those of shUSP29.

### *In vitro* deubiquitination assay.

Ubiquitinated ORF9b was immunoprecipitated from HEK293T cells transfected with expression vectors of ORF9b-GFP, Ub-Flag using an anti-GFP antibody-conjugated protein G agarose beads. USP29 or its mutant was immunoprecipitated from HEK293T cells overexpressing USP29-Myc or its mutant, using anti-Myc antibody-conjugated protein G agarose beads. *In vitro* deubiquitination assay, ubiquitinated ORF9b was incubated with purified USP29 or its mutant in deubiquitination buffer (20 mM Tris-HCl, pH 8.0, 200 mM NaCl, 1 mM EDTA, 10 mM DTT, and 5% glycerol) for 12 h at 37°C. Ubiquitinated ORF9b was analyzed by immunoblotting.

### Statistical analysis.

All data represent the results of three independent experiments and are presented as the mean ± standard deviation (SDs). Differences among groups were analyzed using ANOVA. NS, not significant; *, *P *< 0.05; **, *P *< 0.01; ***, *P *< 0.001; ****, *P *< 0.0001).
