# Supplementary figures and images for: The Deubiquitinase USP29 Promotes SARS-CoV-2 Virulence by Preventing Proteasome Degradation of ORF9b
Source: mBio. 2022 May 31;13(3):e01300-22. doi: 10.1128/mbio.01300-22 (PMC9239186; doi:10.1128/mbio.01300-22)

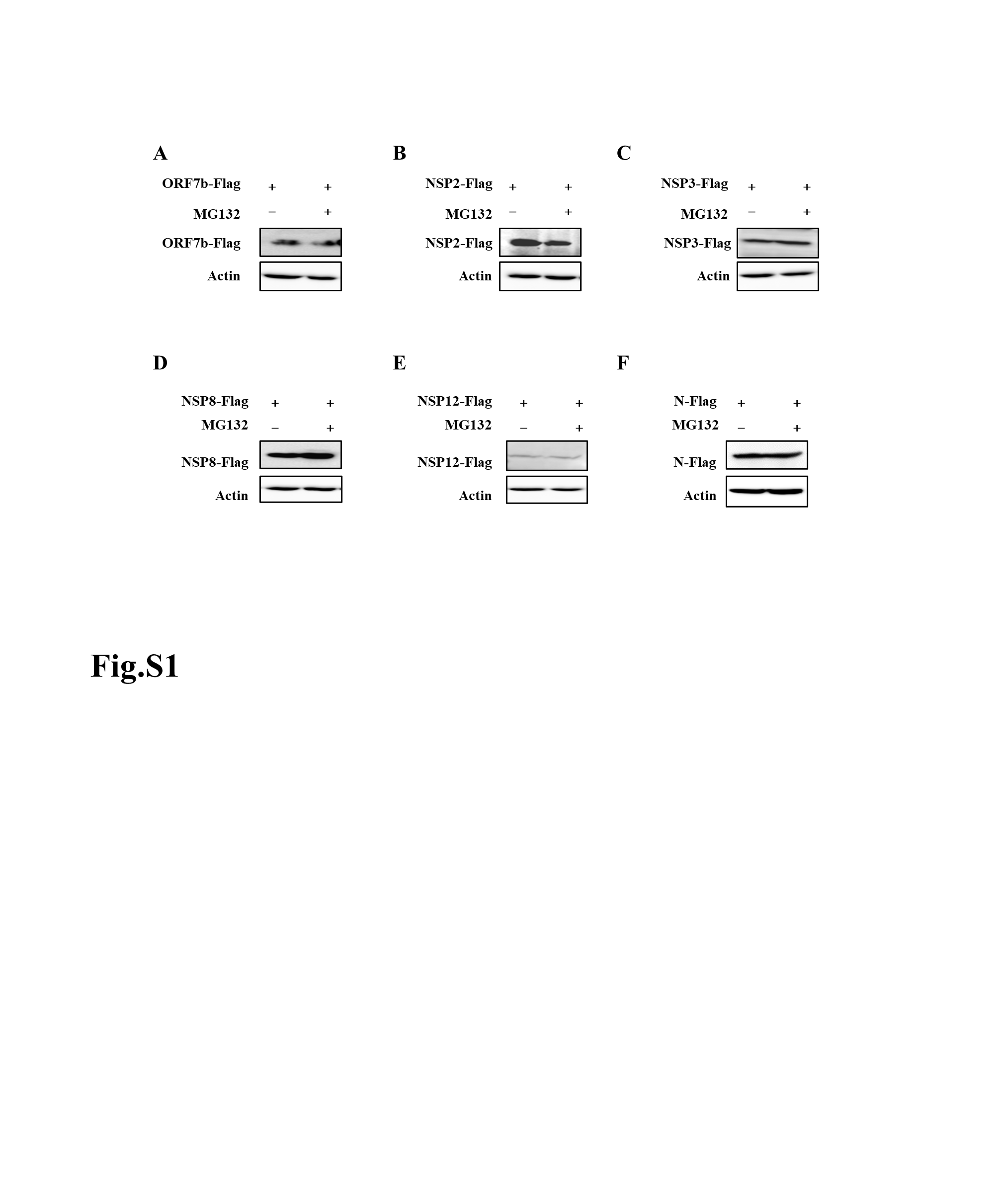

Supplement: FIG S1 [file mbio.01300-22-s0001.tif]

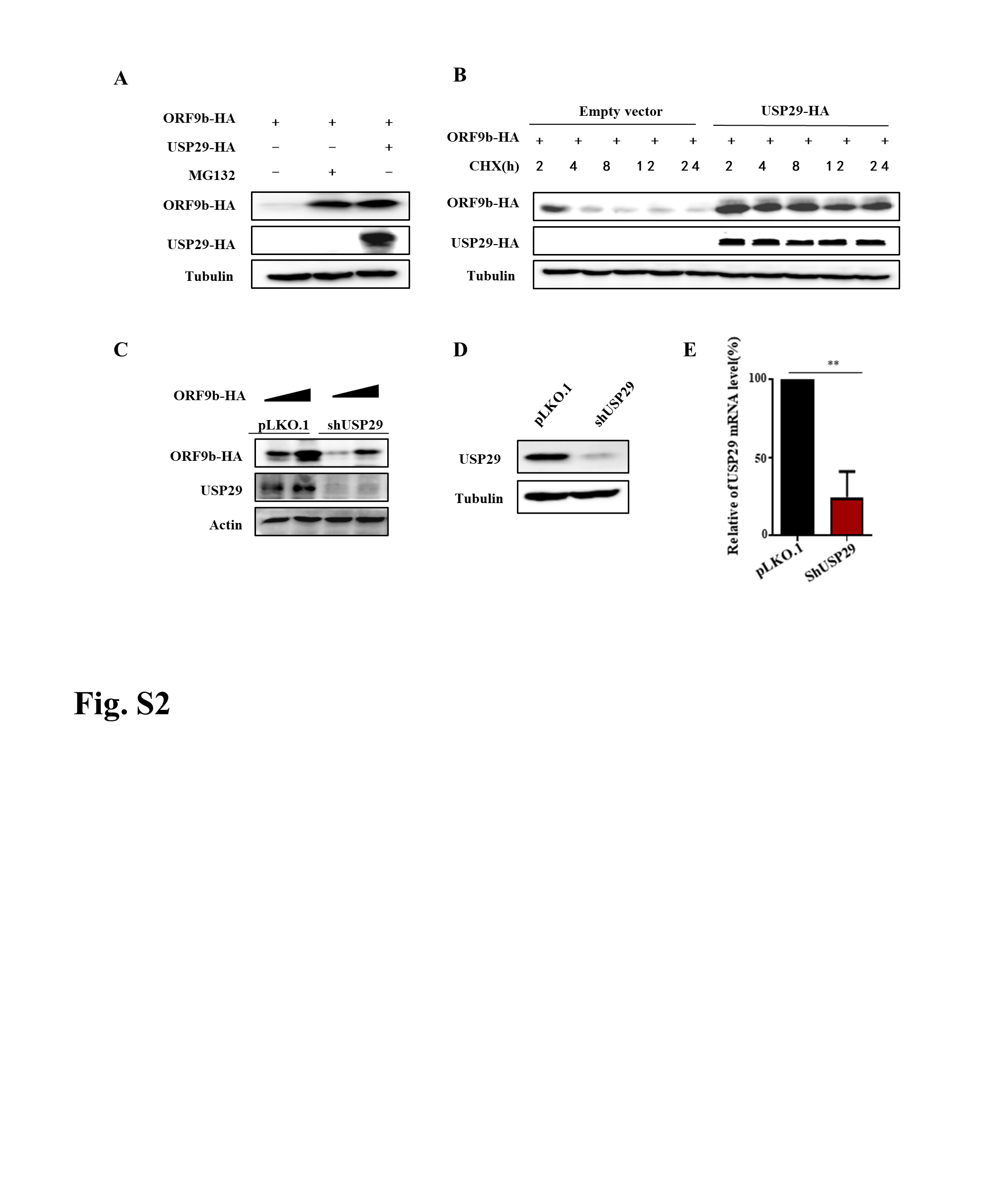

Supplement: FIG S2 [file mbio.01300-22-s0002.tif]

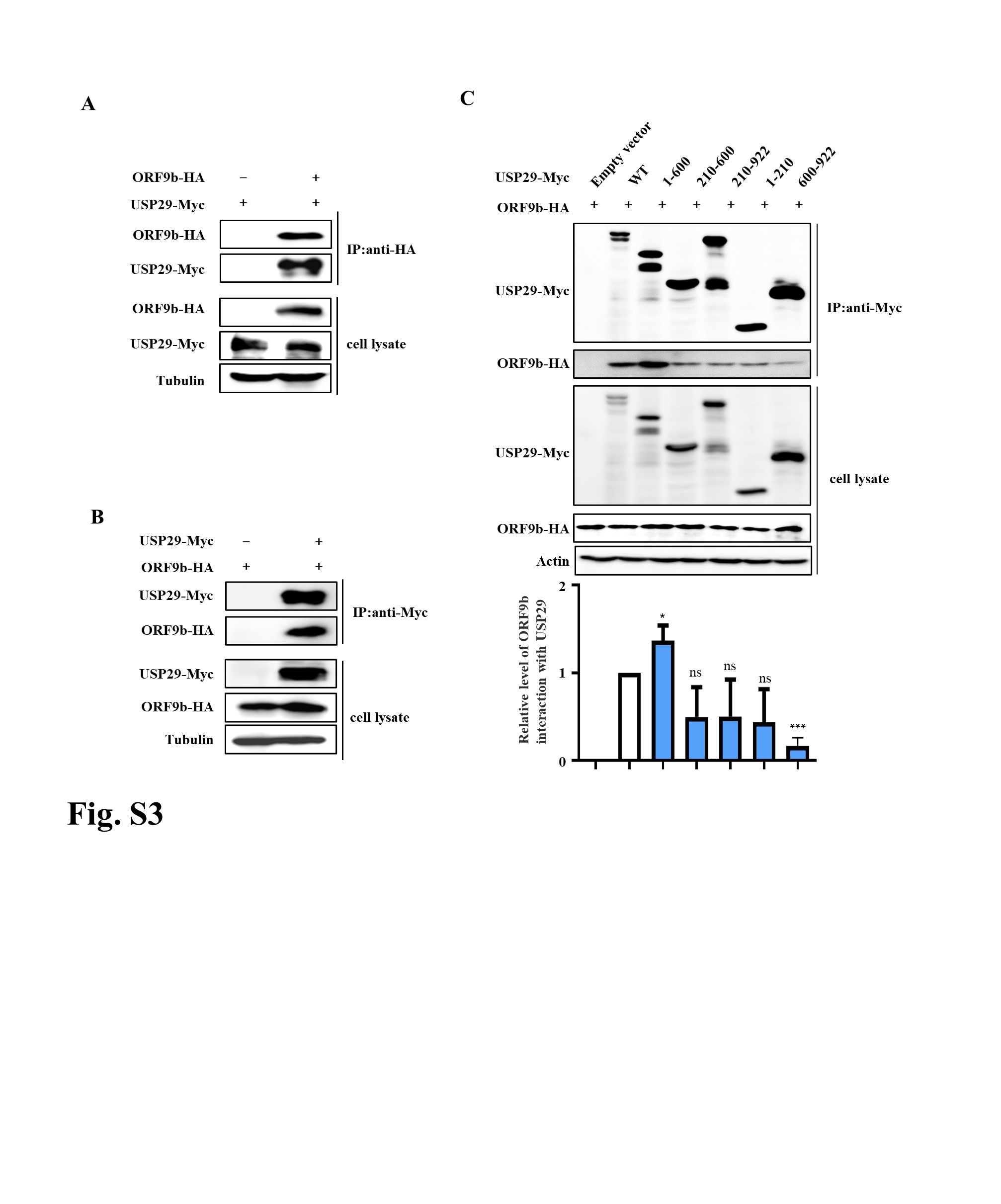

Supplement: FIG S3 [file mbio.01300-22-s0003.tif]

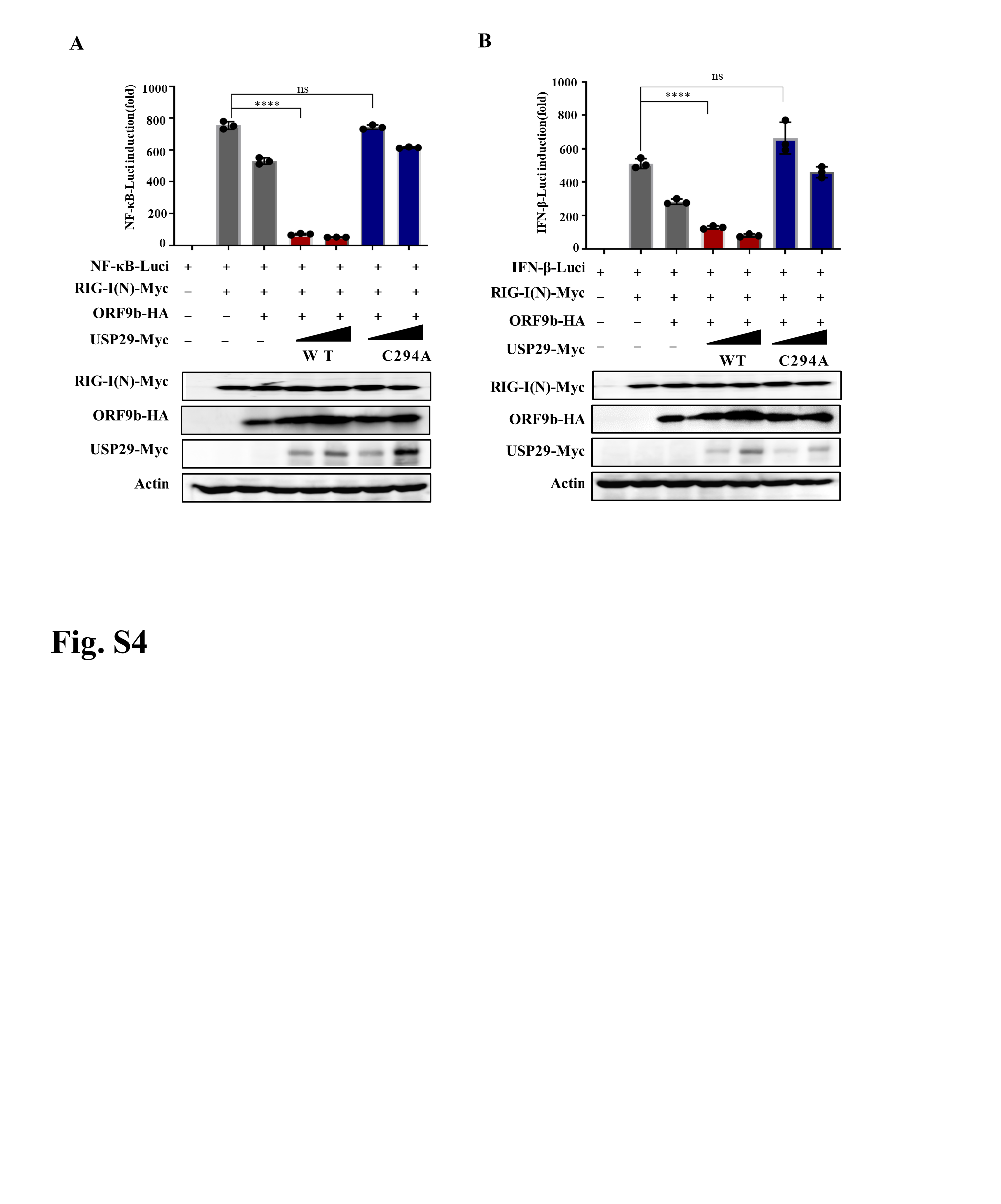

Supplement: FIG S4 [file mbio.01300-22-s0004.tif]

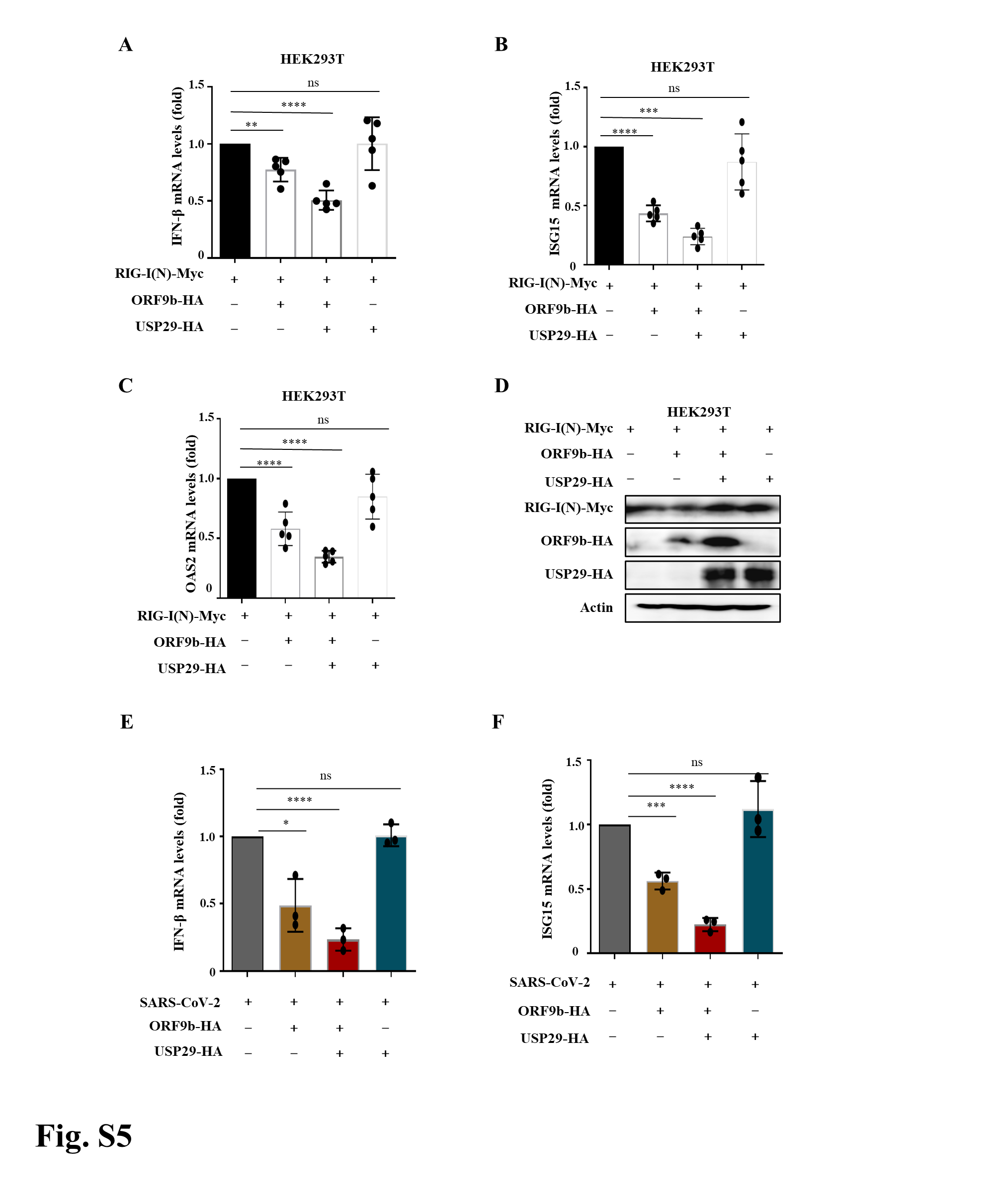

Supplement: FIG S5 [file mbio.01300-22-s0005.tif]
